# Mucosal immune and stress responses of *Neoparamoeba perurans*-infected Atlantic salmon (*Salmo salar*) treated with peracetic acid shed light on the host-parasite-oxidant interactions

**DOI:** 10.3389/fimmu.2022.948897

**Published:** 2022-08-25

**Authors:** Carlo C. Lazado, David A. Strand, Mette W. Breiland, Francisco Furtado, Gerrit Timmerhaus, Mona C. Gjessing, Sigurd Hytterød, Grigory V. Merkin, Lars-Flemming Pedersen, Karin A. Pittman, Aleksei Krasnov

**Affiliations:** ^1^ Nofima, The Norwegian Institute of Food, Fisheries and Aquaculture Research, Ås, Norway; ^2^ Norwegian Veterinary Institute, Ås, Norway; ^3^ Nofima, The Norwegian Institute of Food, Fisheries and Aquaculture Research, Tromsø, Norway; ^4^ Centre for Interdisciplinary Research in Animal Health (CIISA), Faculty of Veterinary Medicine, University of Lisbon, Lisbon, Portugal; ^5^ Quantidoc AS, Bergen, Norway; ^6^ DTU Aqua, Section for Aquaculture, The North Sea Research Centre, Technical University of Denmark, Hirtshals, Denmark; ^7^ Department of Biological Sciences, University of Bergen, Bergen, Norway

**Keywords:** amoebic gill disease, aquaculture, fish health, gill health, mucosal immunity, mucous cells, oxidative stress, parasitic infection

## Abstract

Treatment development for parasitic infestation is often limited to disease resolution as an endpoint response, and physiological and immunological consequences are not thoroughly considered. Here, we report the impact of exposing Atlantic salmon affected with amoebic gill disease (AGD) to peracetic acid (PAA), an oxidative chemotherapeutic. AGD-affected fish were treated with PAA either by exposing them to 5 ppm for 30 min or 10 ppm for 15 min. Unexposed fish from both infected and uninfected groups were also included. Samples for molecular, biochemical, and histological evaluations were collected at 24 h, 2 weeks, and 4 weeks post-treatment. Behavioral changes were observed during PAA exposure, and post-treatment mortality was higher in the infected and PAA treated groups, especially in 10 ppm for 15 min. Plasma indicators showed that liver health was affected by AGD, though PAA treatment did not exacerbate the infection-related changes. Transcriptome profiling in the gills showed significant changes, triggered by AGD and PAA treatments, and the effects of PAA were more notable 24 h after treatment. Genes related to immune pathways of B- and T- cells and protein synthesis and metabolism were downregulated, where the magnitude was more remarkable in 10 ppm for 15 min group. Even though treatment did not fully resolve the pathologies associated with AGD, 5 ppm for 30 min group showed lower parasite load at 4 weeks post-treatment. Mucous cell parameters (i.e., size and density) increased within 24 h post-treatment and were significantly higher at termination, especially in AGD-affected fish, with some treatment effects influenced by the dose of PAA. Infection and treatments resulted in oxidative stress—in the early phase in the gill mucosa, while systemic reactive oxygen species (ROS) dysregulation was evident at the later stage. Infected fish responded to elevated circulating ROS by increasing antioxidant production. Exposing the fish to a crowding stress revealed the interference in the post-stress responses. Lower cortisol response was displayed by AGD-affected groups. Collectively, the study established that PAA, within the evaluated treatment protocols, could not provide a convincing treatment resolution and, thus, requires further optimization. Nonetheless, PAA treatment altered the mucosal immune and stress responses of AGD-affected Atlantic salmon, shedding light on the host-parasite-treatment interactions.

## 1 Introduction

Fish gills are multifunctional organs and play a fundamental role in gas exchange, ion regulation, osmoregulation, acid-base balance, ammonia excretion, and hormone production (1). With its close contact with the aquatic environment, they are portals and attachment sites for many pathogens and as such, exhibit a wide range of immune defense mechanisms (2). In Atlantic salmon (*Salmo salar*) farming, gill health is presently considered as one of the major production problems worldwide (3). Numerous physical, chemical, and biological factors can affect gill health on the farms, especially in sea cages where risks are higher compared with land-based closed systems; one of which, amoebic gill disease (AGD), is a long-standing challenge that dates back to the 80s (4, 5). AGD is caused by the parasite *Neoparamoeba perurans*, initially identified in Tasmania, Australia but now found in different parts of the globe where salmon is being farmed. In Norway, the first documented case of AGD in farmed salmon was in 2006, and the report suggested that the marine environment was a reservoir for the amoeba (6). The colonization of the gills by the parasite initiates a tri-phasic host response that includes a localized reaction to parasite attachment, non-specific immuno-regulatory cell infiltration and advanced hyperplasia with epithelial stratification (7). Gross pathology is commonly assessed by gill scoring based on the severity and prevalence of white mucoid patches on the gill surface (8, 9), and, histologically, lesions are characterized by hyperplasia, lamellar fusion, lamellar vesicles in addition to the presence of amoeba (10, 11). The increasingly common mandatory delousing in commercial salmon farms can cause damage to the gills and other mucosal barriers, damage which persists up to 3 weeks post-treatment and leaves the fish vulnerable to pathogens; reduced growth is likewise observed during this period (12). Although mortality is often considered a moderate problem associated with AGD, it is a significant cause of compromised welfare and reduced growth. These consequences, if not addressed, will serve as contributing factors to other serious health problems (13, 14). According to the Norwegian Fish Health Report (15), the number of AGD outbreaks and the degree of severity in Norwegian salmon farms varies from year to year.

AGD initiates immunological responses in the gill mucosa, but changes can also be observed at the systemic level (11, 16, 17). Mucins are glycopolymers of mucus, and the expression of two mucins (*muc5*, *muc18*) showed differential regulation in AGD-affected fish where upregulation of the secreted *muc5* was detected, while the membrane bound *muc18* demonstrated downregulation (16). A whole-genome wide transcriptomic studies revealed that the early onset of disease is characterized by the activation of complement and the acute phase proteins and simultaneous immunosuppression particularly in the cytokine signaling pathway (5). However, a number of targeted qPCR studies on cytokine expression during AGD infection showed contradictory results, which was partly explained by a number of factors during experimental infection, including the stage of AGD lesion, infectious dose, and sampling time among many others (11). At a later stage of infection, it was shown that genes involved in the major histocompatibility complex I (MHC-I) pathway were substantially downregulated, and the downregulation of *interferon regulatory factor 1* (*irf1*) has been implicated to mediate this response (18). AGD changed the expression of antigen presenting cells, including B cells and T cells. Particularly, CD8+ cells and not CD4+ T cells primarily constituted the T-cell response (19). Furthermore, it was demonstrated that the genes regulating the T helper subsets including Th1 (*ifnγ, tnfα3*), Th17 (*il17a/f1b, il17d, il22*), and Treg (*tgfβ1b, il10a, il10b*) were significantly downregulated, whereas Th2 pathway (il4/13a, il4/13b) was significantly upregulated at the later stage of infection (20).

With the unavailability of vaccines, AGD management relies on therapeutic interventions. Freshwater bath treatment is the most common therapeutic method for AGD and, by far, the only method that provides favorable treatment results (9, 21). Even though this method is effective in controlling AGD to a greater extent, the strategy entails significant infrastructure costs and is labor expensive. One important consideration that remains a significant challenge is the requirement for a nearby freshwater source (22). Oxidative disinfectants are often used against many ectoparasites in fish (23) and, likewise, have been explored for the treatment of AGD. Hydrogen peroxide (H_2_O_2_) was identified to affect AGD and was regarded as a preferred method when freshwater accessibility poses a daunting logistical challenge (24), although the H_2_O_2_ treatment procedure induces persistent damage to all exposed fish mucosa (12). H_2_O_2_ effectiveness is found to be less than freshwater bathing, although both treatments are not fully effective because AGD was observed to re-appear in treated fish, both under natural and controlled experimental conditions (9, 21). Moreover, issues on the H_2_O_2_ toxicity towards non-target organisms, such as shrimps, have been highlighted in recent years and, thus, raise significant concern about H_2_O_2_ use, particularly in Norwegian aquaculture (25). Therefore, new chemotherapies for AGD that are effective and pose minimal risk on fish health and environment must be explored.

In the present study, we evaluated peracetic acid (PAA), a potent oxidant commercially available as an acidified mixture of acetate and H_2_O_2_ (26), as a candidate treatment for AGD. PAA has been shown to have a broad spectrum of antimicrobial activity (23, 27), and its biocidal action is underscored by denaturation of protein, disruption of cell-wall permeability, and oxidation of sulfhydryl and sulphur bonds in proteins, enzymes, and other metabolites (28). Toxicity is often a major issue in using PAA in fish; nonetheless, the effective dose for many aquaculture pathogens is low compared with H_2_O_2_ (29). In a series of studies, we have identified the health and welfare consequences of using PAA (e.g. 0.6 mg/L to 10 mg/L) in Atlantic salmon smolts and found the range of doses that carries minimal risks, at least for naïve, uninfected fish (30–33). Physiological responses of smolts to PAA are not only influenced by dose but also by frequency of application, size of fish, and stress status. PAA, as an oxidant, has been demonstrated to be a potent trigger of oxidative stress and induced structural alterations in the mucosa. Nonetheless, salmon smolts orchestrated a cascade of mucosal and systemic adaptive responses to counteract the physiological pressures from PAA exposure (32, 33). Here, we reported the first study that employed PAA as a candidate treatment for parasitic infection in Atlantic salmon. Using an array of response parameters—behavior, visual evaluation, histology, biochemical analysis, and gene expression profiling, we identified the effects of AGD on host physiology and elucidated how the oxidative chemotherapeutics regulated these responses that could influence potential disease resolution.

## 2 Materials and methods

### 2.1 Ethical considerations

The study adhered to the Guidelines of the European Union (Directive 2010/63/EU) and the Norwegian Animal Welfare Act and was approved by the Norwegian Food Safety Authority under FOTS ID 20/37233. Key personnel of the trial hold a FELASA C Certificate.

### 2.2 Induction of amoebic gill disease in Atlantic salmon smolts

The fish experiment was performed at the Fish Health Laboratory of Tromsø Aquaculture Research Station (HiT), Norway. Atlantic salmon eggs were purchased from a commercial supplier and reared at the station under normal production protocol until they reached the experimental size of around 80–90 g. Fish were screened for relevant bacterial, viral, and parasitic pathogens before commencing the trial. There were 720 fish at the start of the trial. They were divided into two groups—one group was the “uninfected” group, whereas the other group was the “infected” group. Each group had 360 fish stocked in 1 800-L tank. AGD was induced in the infected group by exposing the fish to *Neoparamoeba perurans*: the isolate was from an outbreak in South-West Norway during Autumn of 2019 (17). The pathogenicity of the isolate was established earlier in a pre-trial. Experimental infection was induced as follows: water flow was closed, the amoeba culture was added directly to the tank achieving a concentration of 1 500 parasites per L, and fish were exposed for 1 h. During the exposure period, the level of oxygen was routinely monitored to ensure that the dissolved oxygen (DO) level did not go below 85% saturation. After the exposure period, water was immediately flushed out and replaced with clean water. The fish from the uninfected group were handled similarly but without the addition of the parasite. The experimental fish were under the following conditions: water flow rate in the tanks was 6-7 L/min, the water temperature at 14.5°C, oxygen at >85% saturation, salinity at 35 ppt, photoperiod set at 24 light: 0 darkness, and continuous feeding regime with a commercial diet (Skretting Nutra Olympic 3 mm, Averøy, Norway) administered through a belt feeder. These conditions were likewise adapted throughout the trial.

### 2.3 Oxidant treatment by PAA

**Figure 1:**
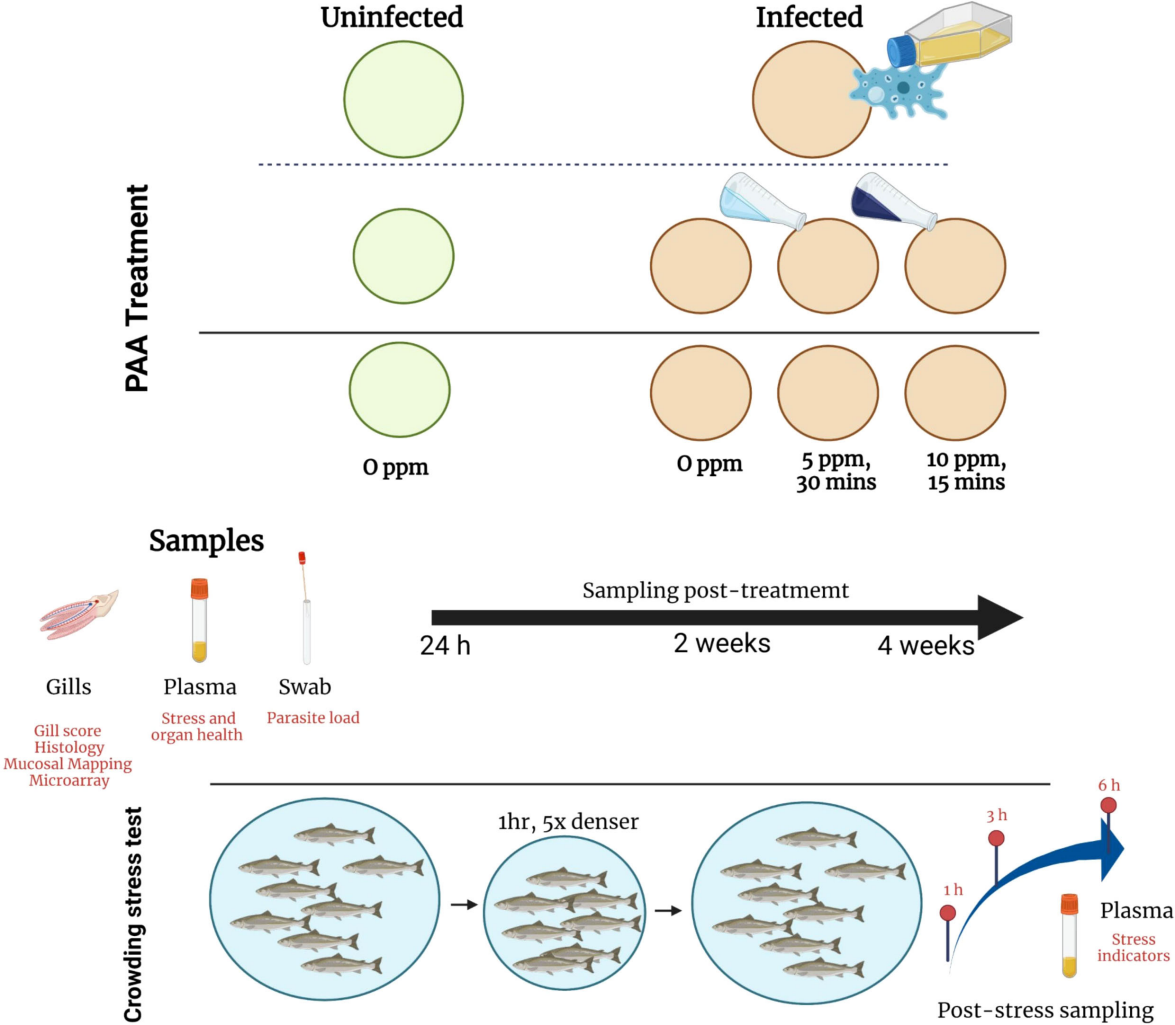
Experimental set-up: distribution of treatment groups and sampling strategies. AGD was induced by bath exposure to *N. perurans*. PAA treatment was performed by exposing the infected fish to 5ppm PAA for 30 min or 10 ppm for 15 min. Uninfected–0 ppm group served as control. Sampling was performed at three occasions. After the last sampling, fish were exposed to a crowding stress test. Created with BioRender.com (Agreement number VF2434DFB9).

Twenty (20) days after infection, fish were distributed to 8 500-L exposure tanks, four tanks for untreated and four tanks for treated groups (**Figure 1**). Each tank was stocked with 60 fish per tank. Fish were allowed to acclimate for 5 days before the PAA treatment was performed. The exposure protocol was as follows: Water flow in the tank was closed. Divosan Forte VT6 (Lilleborg AS, Olso, Norway), a commercially available PAA product, was added to the tank at several locations to achieve the final concentration of either 5 or 10 ppm. Aeration was provided to allow mixing. The exposure protocol had been previously standardized (32). Before the treatment, the actual PAA and hydrogen peroxide concentration in the trade product was analytically verified by DTU Aqua. The fish group treated with 5 ppm was exposed for 30 mins, whereas the 10 ppm group was exposed for 15 min. These concentrations and exposure durations were earlier identified to pose moderate health risks to naïve Atlantic salmon smolts (31, 32). In addition, these concentrations were found to exhibit amoebicidal activity against *N. perurans in vitro* (unpublished data). During the exposure period, fish behavior was documented by trained personnel who recorded changes every 5 min. Some of the behavioral changes that were routinely noted include: erratic swimming, loss of balance, rapid opercular ventilation, and clustering near the water inlet. Oxygen was supplied during exposure to ensure that DO was >85% saturation. After the exposure period, water flow was opened, and water was replaced to at least 75% in the next 8-10 min (**Figure 1**).

### 2.4 Sample collection

The sampling outline is presented in **Figure 1**. There were three major tissue samplings—24 h, 2 weeks, and 4 weeks after the treatments. For each sampling occasion, five fish were randomly netted from each tank and humanely euthanized with a bath overdose of Benzoak Vet (ACD Pharmaceuticals AS, Leknes, Norway). The fork length and weight of the fish were recorded. Blood was collected using lithium heparinized vacutainer (BD, Plymouth, United Kingdom) from the caudal artery, centrifugated for 10 min at 5,200 rpm (Heraeus Labofuge 200, Thermo Scientific, Massachusetts, USA), plasma was separated and stored at -80°C until analysis. After the macroscopic gill score was assessed (8), gill swabs (Sarstedt, Germany) were taken from the left side of the gills and stored in ATL buffer (Qiagen, Hilden, Germany) for the qPCR quantification of parasite load. The first gill arch of the right side of the fish was collected and suspended in 10% neutral buffered formalin (BiopSafe^®^, Stenløse, Denmark) for Quantidoc’s mucosal mapping analysis. The second gill arch was, likewise, dissected and divided into two—one fraction (i.e., non-lesion region) was placed in RNAlater (Ambion^©^, Connecticut, USA), kept at room temperature for 12 h for penetration and afterwards stored at -80°C until further use, whereas the other fraction was stored in 10% neutral buffered formalin (BiopSafe^®^, Stenløse, Denmark) for histological use.

### 2.5 Crowding stress

Salmon are often exposed to different production-related stress. Infection and treatment are factors that could influence the ability of the fish to respond to secondary stressors, therefore, we explored whether AGD and PAA treatment could impact the innate stress responses of salmon. Twenty four (24) h after the last tissue sampling, the remaining fish (N=30 per replicate tank) were subjected to a crowding stress test by lowering the water volume in the tank to achieve a density 5x higher than the initial density. Fish were under this condition for 1 h, thereafter, the water level was returned to the initial level. DO level was maintained at >85% saturation and, whenever necessary, oxygen was injected during the crowding. Five fish at 1, 3, and 6 h were sampled from each tank after the stress challenge for plasma collection. Euthanasia and sample handling were similar, as described in Section 2.4.

### 2.6 Plasma stress and organ health indicators

Pentra C400 Clinical Chemistry Analyzer (HORIBA ABX SAS, Montpellier, France) was used to determine the level of lactate, glucose, alkaline phosphatase (ALP), alanine aminotransferase (ALAT), lactate dehydrogenase (LDH), and creatinine (CR) in plasma. Commercially available kits were used to measure cortisol (Demeditic Diagnostics GmbH, Kiel, Germany), total antioxidant capacity (TAC, Sigma Aldrich, Missouri, USA), and reactive oxygen species (ROS)/reactive nitrogen species (RNS) (CellBiolabs, Inc., California, USA) in plasma. The TAC kit measures the concentration of the combination of both small molecule and protein antioxidants, and TOC is expressed as Trolox (a water-soluble vitamin E analogue) equivalent. The ROS/RNS kit, a collection of ROS and RNS including hydrogen peroxide, nitric oxide, peroxyl radical, and peroxynitrite anion, uses a proprietary fluorogenic probe, DCFH-DiOxyQ. Lactate, glucose and cortisol are key markers for stress level, ALP and ALAT are indicators of liver health, LDH is an indicator of tissue damage, CR accounts for kidney function, and TAC and ROS provide information about oxidative stress level. All analyses were performed in duplicate.

### 2.7 RNA isolation and microarray analysis

Total RNA from the gills (N=6 fish per treatment group; three per replicate tank) was isolated from the RNAlater^®^-preserved samples using the Agencourt RNAdvance™ Tissue Total RNA Purification Kit (Beckman Coulter Inc., CA, USA). All samples had an RNA Integrity Number (RIN) above 8.4 as evaluated by the Agilent^®^ 2100 Bioanalyzer™ RNA 6000 Nano Kit (Agilent Technology Inc., Santa Clara, CA, USA). The microarray analysis was performed using a custom-designed 15K Atlantic salmon DNA oligonucleotide microarray SIQ-6 (Agilent Array, ICSASG_v2), and all re-agents that were used were from Agilent Technologies. The One-Color Quick Amp Labelling Kit (CA, USA) was used for RNA amplification and Cy3 labelling using 110 ng of RNA template per reaction. Gene Expression Hybridization Kits were used for the fragmentation of labelled RNA. This was followed by a 15-h hybridization in a 65°C oven with a constant rotational speed of 10 rpm. Thereafter, the arrays were successively washed with Gene Expression Wash Buffers 1 and 2 and scanned using the Agilent SureScan Microarray Scanner. Pre-processing was performed in Nofima’s bioinformatics package STARS (Salmon and Trout Annotated Reference Sequences) (34).

### 2.8 Parasite load quantification

DNA was extracted from the gill swabs using the DNeasy Blood and Tissue Kit (Qiagen, Hilden, Germany). The DNA samples were analyzed using a *N. perurans* specific qPCR to confirm the presence of *N. perurans* and estimate DNA copies as a measure of parasite load (17). The samples were analyzed on the CFX96 Touch System (Biorad, California, USA) with 25 µl reactions consisting of 12.5 µl TaqPath qPCR Mastermix, 500 nM of each primer and 250 nM of probe (forward primer 5’-GTT CTT TCG GGA GCT GGG AG-3’, reverse primer 5’- CAT GAT TCA CCA TAT GTT AAA TTT CC-3’ and probe 5’-FAM/CTC CGA AAA/ZEN/GAA TGG CAT TGG CTT TTG A/3IABkFQ-3’), PCR grade water, and 5 µl DNA sample. The thermocycling conditions were as follows: an initial denaturation at 95°C for 20 s, followed by 50 cycles of denaturation at 95°C for 3 s and annealing at 60°C for 30 s. In each qPCR run, a 10-fold standard dilution series were included in order to estimate DNA copies per sample reaction. The standard dilution consisted of synthesized dsDNA (gBlocks™ gene fragment, Integrated DNA Technologies, Iowa, USA) of the qPCR target region with known DNA concentrations.

### 2.9 Histological evaluation

Formalin-preserved gills were processed, and sections were stained with Haematoxylin-Eosin and digitized by the Pathology Division of the Norwegian Veterinary Institute in Harstad, Norway. Histological assessment of the gills was carried out with semi-quantitative scoring (0–5) of the following parameters: epithelial hyperplasia as seen in AGD (segmental hyperplasia dominated by epithelial cells), other epithelial hyperplasia, and lesions in lamellar vessels. The presence of amoeba, epitheliocysts, was noted as recorded or not recorded.

### 2.10 Mucosal mapping

The formalin-preserved gill samples were subjected to Mucosal Mapping following the standard protocol of Quantidoc AS (35). Mucosal Mapping describes the design-based stereological quantitative measurement of mucous cell sizes, mucous cell volumetric densities in the epithelium, rather than numeric density and their abundance in mucosal barriers of skin, gills, and intestines. Tissue sections stained by Periodic Acid Schiff – Alcian Blue were digitized, scanned, and processed through an automated software developed by Quantidoc AS for the stereological image analysis of the gill mucosa (Veribarr™). The analyzed mucosal features include mucous cell volumetric density (D, % epithelium filled with mucous cells) and mean mucous cell area at the cell equator (A, μm²) (35–37).

### 2.11 Data handling

All statistical tests were performed in SigmaPlot (Systat Software Inc., London, UK), except for microarray data. The normal distribution was evaluated using a Shapiro-Wilk test, whereas equal variance was analyzed by a Brown-Forsythe test before a two-way analysis of variance (ANOVA), which was employed to assess differences within treatment groups and timepoints. When at least one of the ANOVA requirements was not met, the dataset was log-transformed before performing the analysis. The statistical level of variance was set at *p* < 0.05. All data are presented as mean ± standard deviation (S.D.).

For mucous cell parameters, a Linear mixed effect model (lme) was used to test differences among treatments and sampling points (R studio, Massachusetts, USA). Statistical significance was set at P ≤ 0.05.

The microarray results were exported from STARS as log2 transformed expression ratios (ER) and further processed in R (version 4.0.2, https://www.r-project.org/). Expression levels were normalized to the mean expression after 24 h, untreated and uninfected groups. Significant differential expressed genes (DEGs) were defined by (1) P-value cut-off of <0.05 (ANOVA, aov() function, *stats* package) for any of the three factors, timepoint (three levels), treatment (two levels), and AGD infection (two levels). (2) Group means were calculated for 12 subgroups, and a minimum difference of >1 between the lowest and the highest mean was used to exclude genes with low effect-sizes. Genes that fulfilled both requirements (1) and (2) were defined as DEGs. This resulted in 1,054 DEGs, which were represented in a heatmap (heatmap.2() function, *gplots* package, **Figure 3**). Distances between genes were calculated using the Euclidean distance method, and the dendrogram was calculated by a complete linkage algorithm. The dendrogram was split into 12 clusters with distinctive expression patterns. Four clusters with similar expression patterns contained only one or two genes and were combined into a single cluster for presentation. The functional annotation terms, as they are used in STARS, where tested for significant enrichment within these clusters (fisher.test() with alternative hypothesis set to “greater” only, *stats* package). Terms with P-values <0.05 are shown next to the heatmap with indication in which cluster they were identified.

## 3 Results

### 3.1 Behavioral changes and performance indicators

Behavioral manifestations in the 5 ppm, 30 min groups during treatment were as follows: 1) the first 5–10 min showed abrupt swimming patterns, typified by avoidance and jumping out of the water; 2) the next 5–10 min were exemplified by rapid opercular ventilation; 3) the last 10 min showed clustering at the bottom of the tank, close to water inlet; opercular ventilation was magnified and not more than 10% of the fish showed loss of balance. For the groups treated with 10 ppm for 15 min, there were two main periods of behavioral changes: 1) The first half of the exposure period was characterized by rapid, erratic swimming and increased opercular ventilation by at least 30% of the population; and 2) the second half showed reduced swimming speed with some fish still showing abrupt swimming, clustering near the water inlet, increased opercular ventilation, and loss of balance in at least 15% of the population. In addition, there was a clear difference between infected and uninfected groups, with the former displaying heightened responses. After 24 h, the survival was as follows: “uninfected–0 ppm” = 100%, “infected–0 ppm” = 100%; “infected–5 ppm, 30 min” = 96.7%; and “infected–10 ppm, 15 min” = 86.7%. No recorded mortality thereafter.

There were no significant differences in weight (average weight 136 ± 12.5 g) and length (23.4 ± 6.2 cm) among the groups at termination.

### 3.2 Organ health indicators in plasma

Two plasma indicators of liver health (i.e., alanine transaminase = ALAT; alkaline phosphatase = ALP) showed significant inter-treatment and temporal differences (**Table 1**). ALAT levels in “infected-0 ppm” and “infected-5ppm,30 min” groups showed significant variations—for the former, a significant decrease in ALAT level at 2 weeks post-treatment, while in the latter, the highest level was identified at 4 weeks post-treatment. The level of ALAT in the “infected-0ppm” group was significantly higher than “uninfected-0ppm” at 24 h and 4 weeks post-treatment. ALP level demonstrated significant temporal variations in all groups, where an increasing tendency over time was predominantly manifested. At 4 weeks post-treatment, the ALP level in all infected groups was significantly higher than the “uninfected-0ppm” group. For creatinine (CR), no significant inter-treatment differences were identified. However, significant temporal changes were observed in the “infected-5 ppm,30 min” and “infected-10 ppm,15 min” groups where the level significantly decreased through time, demonstrating the lowest at 4 weeks post-treatment. There were significant temporal changes in the plasma LDH level. For the “uninfected–0 ppm” group, the levels were significantly higher at 2 and 4 weeks than at 24 h. The LDH level in “infected–0 ppm” was highest at 2 weeks post-treatment and the level was significantly different from 24 h and 4 weeks post-treatment. For the two infected and treated groups: “infected-5 ppm, 30 min” displayed increasing tendency and peaked at 4 weeks post-treatment, whereas “infected–10 ppm, 15 min” group demonstrated significant decrease at 2 weeks post-treatment but returned again to the level at 24 h post-treatment 4 weeks after. At all timepoints, LDH level in “infected–0 ppm” was significantly higher than the “uninfected–0 ppm”. Such a significant difference from the “uninfected–0 ppm” was only observed at 24 h and 4 weeks post-treatment in “infected–5 ppm, 30 min” and “infected–10 ppm,15 min”.

**Table 1:** Plasma indicators of organ health.

Indicator	Treatment	Post-treatment		
		24 h	2 weeks	4 weeks
ALAT (U/L)	*Uninfected–0 ppm*	0.50 ± 0.22	0.79 ± 0.32	1.09 ± 0.19
	*Infected–0 ppm*	1.60 ± 0.31^a*^	0.80 ± 0.63^b^	1.69 ± 0.12^a*^
	*Infected–5 ppm, 30 min*	0.48 ± 0.23^a^	0.66 ± 0.20^a^	1.39 ± 0.21^b^
	*Infected–10 ppm, 15 min*	1.16 ± 0.43	0.95 ± 0.47	1.12 ± 0.29
ALP (U/L)	*Uninfected–0 ppm*	49.1 ± 16.0^a^	116.5 ± 19.5^b^	103.7 ± 22.3^b^
	*Infected–0 ppm*	110.1 ± 19.7^a*^	119.5 ± 18.8^a^	185.2 ± 19.9 ^b*^
	*Infected–5 ppm, 30 min*	70.2 ± 19.9 ^a^	114.1 ± 17.4^b^	162.2 ± 23.5^b*^
	*Infected–10 ppm, 15 min*	73.1 ± 14.8^a^	84.9 ± 23.3^a^	143.0 ± 11.5 ^b*^
CR (U/L)	*Uninfected–0 ppm*	8.05 ± 1.1	6.26 ± 1.29	5.05 ± 2.78
	*Infected–0 ppm*	10.1 ± 2.2	5.63 ± 1.71	2.75 ± 2.32
	*Infected–5 ppm, 30 min*	10.5 ± 1.24^a^	5.91 ± 1.41^a^	3.70 ± 2.71^b^
	*Infected–10 ppm, 15 min*	14.5 ± 4.01^a^	5.91 ± 1.13^b^	4.72 ± 2.53^b^
LDH (U/L)	*Uninfected–0 ppm*	179.7 ± 60.9^a^	345.1 ± 54.8^b^	305.0 ± 45.7^b^
	*Infected–0 ppm*	467.5 ± 93.8^a*^	654.8 ± 43.6 ^b*^	445.5 ± 50.0 ^a*^
	*Infected–5 ppm, 30 min*	317.4 ± 44.4^a*^	310.9 ± 75.2^a^	444.6 ± 62.2^b*^
	*Infected–10 ppm, 15 min*	390.8 ± 70.1^a*^	231.7 ± 51.6^b^	392.2 ± 50.4^a*^

ALAT, alanine aminotransferase; ALP, alkaline phosphatase; CR, creatinine; LDH, lactate dehydrogenase. Different letters denote significant difference over time within a treatment group. Asterisk (*) indicates that the infected group is significantly different from the uninfected group at a particular time point. Values represent mean ± SD of 10 individual fish per group at each sampling point.

### 3.3 Level of ROS and TAC in plasma

**Figure 2:**
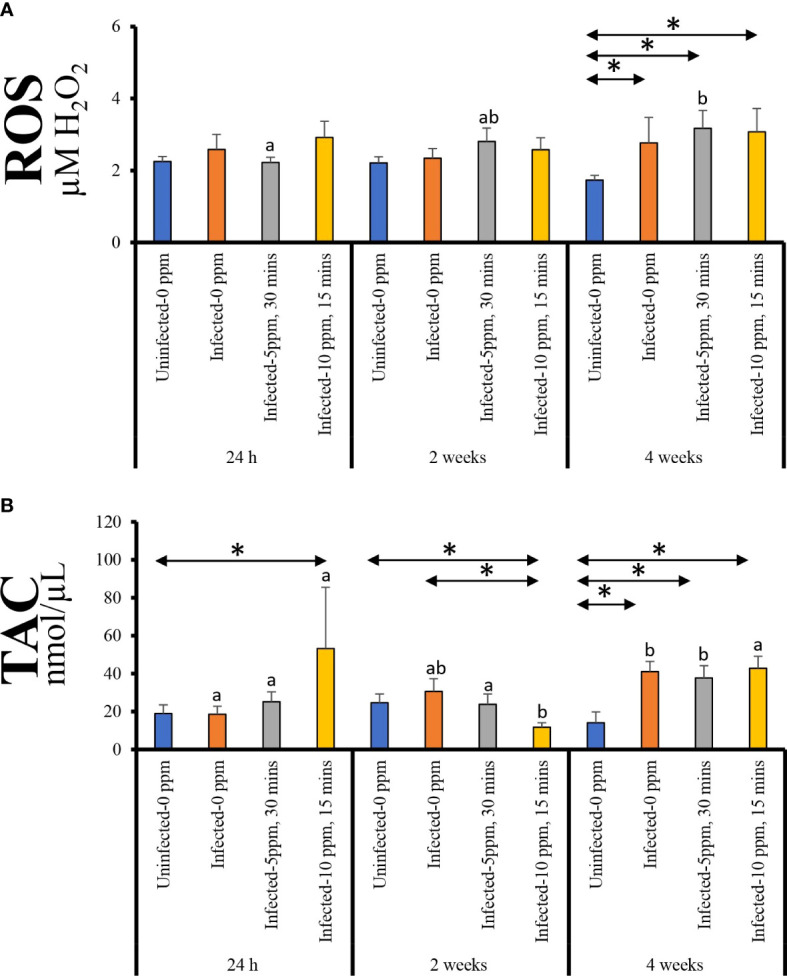
Plasma level of **(A)** reactive oxygen species (ROS, expressed as µM H_2_O_2_) and **(B)** total antioxidant capacity (TAC). Values represent the mean ± SD of 10 individual fish per group at each sampling point. Different letters denote significant differences over time within a treatment group. Asterisk (*) indicates that the two groups at a particular timepoint exhibit significant differences.

Plasma ROS level displayed significant temporal changes only in “infected–5 ppm, 30 min”, where the highest level was observed at 4 weeks post-treatment (**Figure 2A**). Moreover, the ROS level in the three infected groups was significantly higher than the “uninfected–0 ppm” group at this timepoint.

The plasma TAC level showed significant temporal changes in all groups except in “uninfected–0 ppm” (**Figure 2B**). For “infected–0 ppm” and “infected–5 ppm, 30 min” groups, the highest level was observed at 4 weeks post-treatment; at this timepoint, the levels were significantly higher than the “uninfected–0 ppm” group. On the other hand, “infected–10 ppm, 15 min” group demonstrated significantly lower TAC level at 2 weeks post-treatment compared with the two other timepoints. In all timepoints, TAC level in “infected–10 ppm, 15 min” group was significantly different from the “uninfected–0 ppm”—it was significantly higher at 24 h and 4 weeks post-treatment while significantly lower at 2 weeks post-treatment.

### 3.4 Differentially expressed genes in the gills

**Figure 3:**
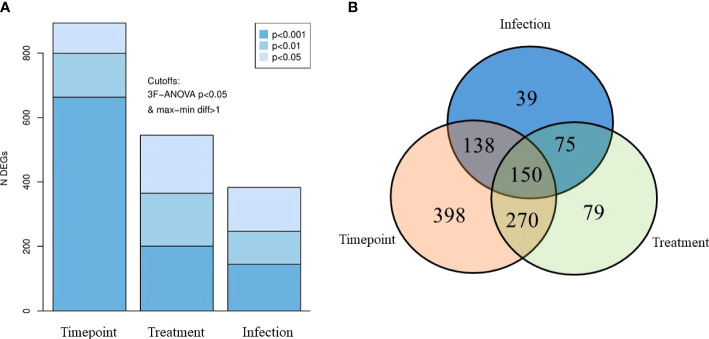
Differentially expressed genes (DEG) in the gills of AGD-affected Atlantic salmon treated with PAA. **(A)** DEGs accounted based on three key factors—timepoint, treatment, and infection status. Shades of blues indicate different P-value levels. **(B)** Venn diagram showing the distribution of the DEGs, with significantly different expressions due to the three factors.

Differentially expressed genes were identified based on the three key factors—timepoints, PAA treatment, and AGD status (**Figure 3A**). Timepoint-wise (i.e., 24 h, 2 weeks, and 4 weeks after treatment), there were 893 DEGs of which 663, 139, and 94 were identified at p value <0.001, <0.01, and <0.05, respectively. Based on PAA treatments (i.e., infected–0 ppm; infected–5 ppm, 30 min; infected–10 ppm, 15 min), 545 DEGs were identified with a distribution of 201, 164, and 180 according to p value <0.001, <0.01, and <0.05, respectively. The AGD factor (i.e., uninfected–0 ppm, infected–0 ppm) had the lowest DEGs at 383, which were distributed as follows: 145 (p <0.001), 102 (p <0.01), and 136 (p <0.05).

Identifying the interactions of these DEGs, we found 150 DEGs common in all factors (**Figure 3B**). There were 398, 79, and 39 DEGs exclusively found in comparisons within timepoints, treatments, and AGD status, respectively. In terms of interaction between two factors, 270 DEGs were found in both timepoint and treatment comparison, while 138 DEGs were identified for timepoint and infection. Seventy-five DEGs were different for infection and treatment.

### 3.5 Functional categories of DEGs in the gills

**Figure 4:**
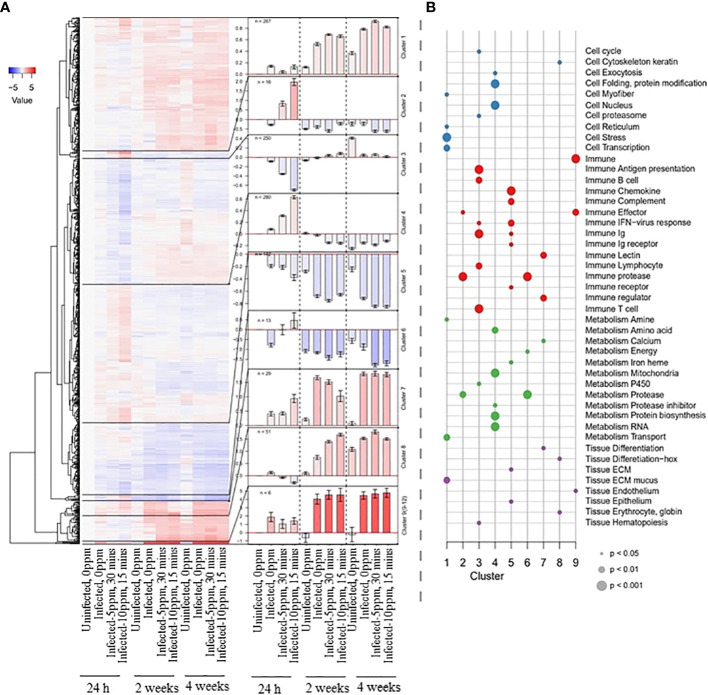
Functional categories of the DEGs identified in the gills of AGD-affected Atlantic salmon treated with PAA. **(A)** The heatmap on the left shows the down- and upregulation of DEGs in a color gradient from blue to red. The dendrogram was split into nine sub-clusters, and the mean values for genes within these clusters are represented in bar plots (error bars show +/- standard error of the mean) in the center. Cluster 9 includes four of the smallest clusters. The color gradient (blue: downregulation; red: upregulation) represents the normalized expression relative to the uninfected–0 ppm group at 24 h after treatment. **(B)** Enrichment analyses of the nine sub-clusters. The identified functional gene categories are shown along the Y-axis, and the nine clusters are arranged along the X-axis. Dots were colored according to the higher categories (cell, immune, metabolism, and tissue), and the size indicates the P-value according to Fisher’s exact test.

We then functionally categorized the DEGs affected by the three factors, which resulted in nine major clusters grouped according to the patterns of their regulation (**Figure 4A**). Cluster 1 is a large cluster consisted of 267 genes. This cluster is categorized as slightly upregulated from 2 weeks post-treatment, particularly in groups affected by AGD. The general response was the induction of cell stress and ECM mucus (**Figure 4B** **;** e.g., *GMP Giant mucus protein, jund, DNA damage-inducible transcript 4 protein-like*). Cluster 2 is relatively a small cluster with 16 genes, characterized by upregulation shortly after PAA treatment. Moreover, the response was stronger at a higher dose, shorter exposure, and primarily involved immune genes (e.g., *C1q and TNF-like domains, leukocyte cell-derived chemotaxin 2-1*). Cluster 3 is a large cluster comprised of 250 genes, represented by down-regulated genes as an early response to PAA treatment. Most of the genes in this cluster include several adaptive immune pathways with B- and T- cells (e.g*., T cell receptor alpha, T cell receptor alpha*). Cluster 4 is the largest cluster with 280 genes. Though large in number, expression of the genes was weakly upregulated as an early response to PAA and represented mainly by protein synthesis and metabolism-related genes (e.g., *glutamine synthetase, heat shock protein 70*). Cluster 5 is also a relatively large cluster with 142 down-regulated genes, where the most substantial effect was observed in AGD affected groups regardless of the treatment at 2 and 4 weeks. The group contains mainly innate and early immune response genes (e.g., *GTPase IMAP family member 7, interferon-induced guanylate-binding protein 1-like*). Cluster 6 is a small cluster with 13 strongly down-regulated genes, which primarily consisted of Matrix metalloproteinases [e.g*., matrix metalloproteinase-9, matrix metalloproteinase 13 (mmp 13), or collagenase 3*]. At 24 h post-treatment, AGD appeared to repress them, but PAA caused induction. At 4 weeks post-treatment, the strongest repression was in the PAA treated groups. Cluster 7 comprises of 29 genes with a strong up-regulation in AGD affected groups. There was a different regulation in “infected–10 ppm, 15 min” group at 24 h (higher than the other AGD groups) and 2 weeks after treatment (lower than the other AGD groups). The group includes several immune signaling genes (e.g., *regulator of G-protein signaling 21-like, arginase, type II*). Cluster 8 contains 51 strongly upregulated genes at the later timepoints in the AGD group and the “uninfected–0 ppm” group. At 2 weeks post-treatment, a stronger expression was characterized in the groups exposed to PAA. The last group, Cluster 9 is a combined cluster of four small clusters and contains six genes. These genes were strongly upregulated in all AGD groups. The complete list of DEGs is provided in **Supplementary File 1**.

### 3.6 Gill score, parasite load, histological evaluation, and mucosal mapping

**Figure 5:**
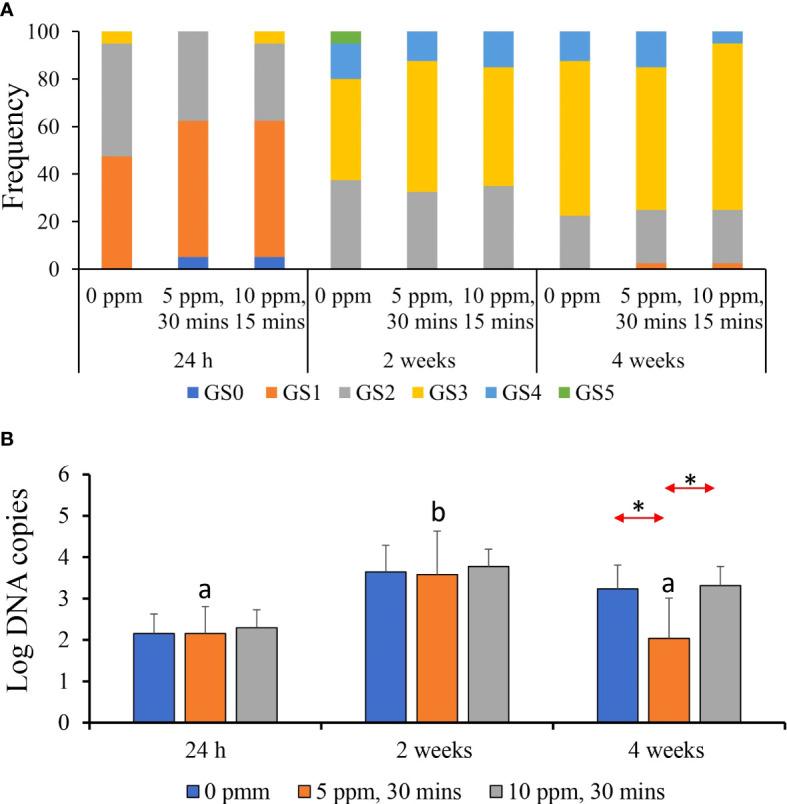
Gross gill scores and parasite load of AGD-affected Atlantic salmon treated with PAA. **(A)** Frequency of occurrence of the gill scores accounted for in each treatment group per timepoint. Each timepoint was represented by 10 individual fish. **(B)** Parasite load in gills quantified by qPCR. Values represent the mean ± SD of 10 individual fish per group at each sampling point. Different letters denote significant differences over time within a treatment group. Asterisk (*) indicates that the two groups at a particular timepoint exhibit significant differences.

At 24 h after treatment, all treatment groups showed an almost identical distribution of gross gill scores (GS)—at least 55%–60% of the evaluated fish exhibited a GS 1, whereas around 25%–30% showed GS 2 (**Figure 5A**). Less than 5% exhibited GS 3. The GS distribution changed at 2- and 4-weeks post-treatment, where an increase to three was evident in all treatment groups. At 2 weeks post-treatment, all groups showed GS 2 in about 40% of the evaluated fish, whereas at least 25%–30% demonstrated GS 3. At 4 weeks post-treatment, GS 3 accounted for about 75%–80% of the evaluated fish. There were no notable inter-treatment differences.

Parasite load from the gill swabs indicated significant temporal difference, especially in 5 ppm–30 min group, where the count was highest at 2 weeks post-treatment (**Figure 5B**). Significant inter-treatment difference was only identified at 4 weeks post-treatment where “infected–5 ppm, 30 min” group had a significantly lower parasite load than the two other groups.

**Figure 6:**
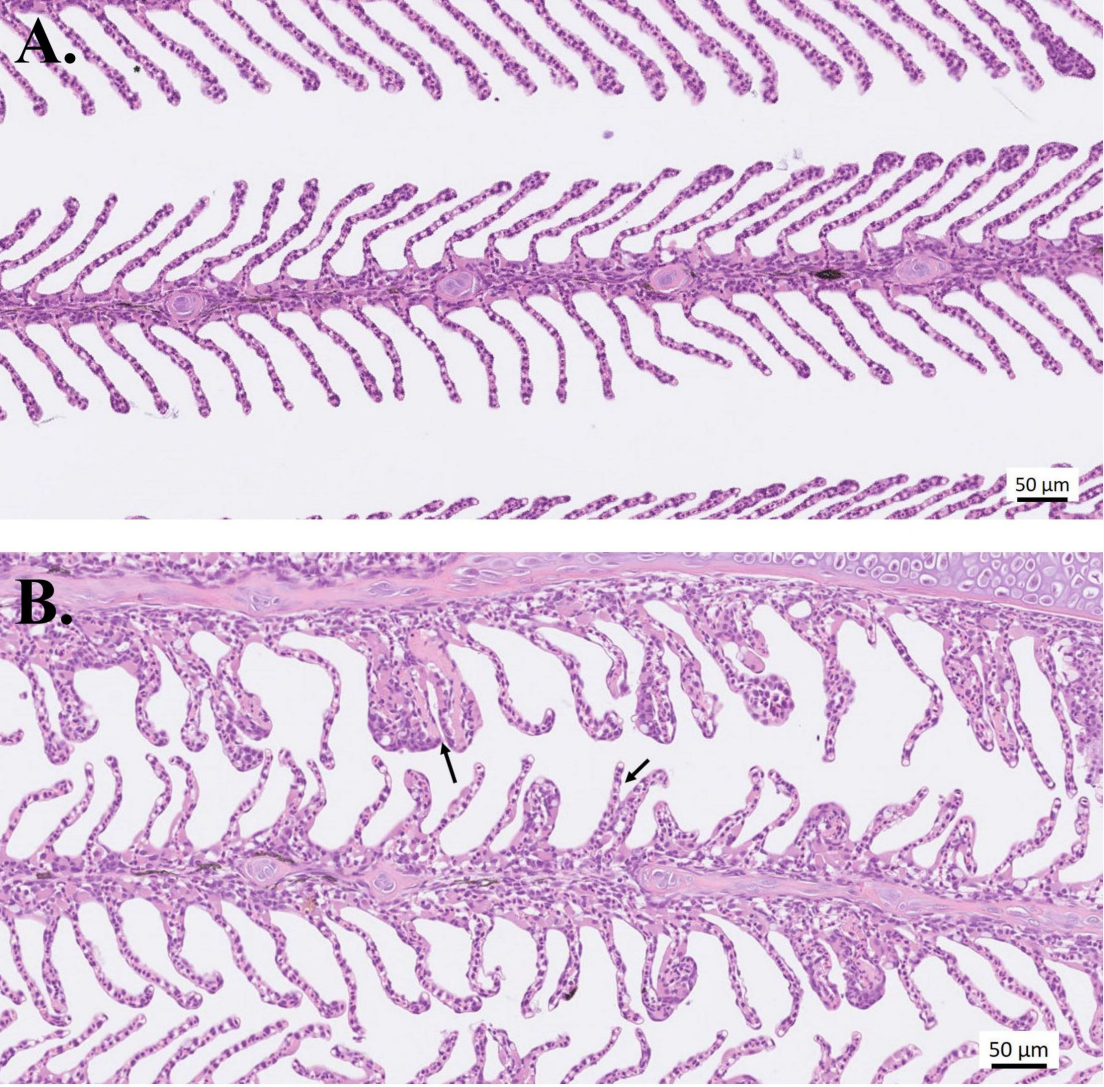
Histological sections of gills stained with H&E. Representative photos of **(A)** Healthy gills from the uninfected–0 ppm group, and **(B)** Lesions in lamellar vessels (left arrow) and lamellar adhesions (right arrow). Infected and treated with 10-ppm PAA for 15 min; sampled 24 h after treatment.

In groups treated with PAA, except for the “infected–5 ppm group”, some lamellar adhesions and moderate to severe lesions in lamellar vessels were found 24 hours after treatment (**Table 2**, **Figure 6**). In the uninfected fish, only mild epithelial hyperplasia not suspected to be AGD related was seen. AGD-related pathologies were likewise evaluated using a 0-to-5–point system and the visual presence of amoeba was recorded as present or not present, as detailed in **Table 2**. At 2 weeks after treatment, 80% of the fish in “infected–0 ppm” group exhibited microscopic AGD pathology scores 2 and higher, 70% in “infected–5 ppm, 30 min” and all evaluated fish in “infected–10 ppm, 15 min”. At 4 weeks after treatment, 100% of the fish in “infected–0 ppm” group exhibited microscopic AGD pathology scores 2 and higher. The group “infected–5 ppm, 30 mins” remained having 80% of the evaluated population with AGD pathology scores 2 and higher, whereas 90% of the evaluated fish in “infected–10 ppm,15 min” showed the same level of severity. The presence of amoeba corresponded to the microscopic GS, which the amount, as visually scored, increased as the disease progressed (**Table 2**). All evaluated fish in “infected–0 ppm” demonstrated microscopic GS 2 and higher at termination. Few epitheliocysts were occasionally seen in all groups during the study.

**Table 2:** Gill histological parameters.

		AGD pathology			Amoeba*		
		Post-treatment			Post-treatment		
	Score	24 h	2 weeks	4 weeks	24 h	2 weeks	4 weeks
Infected*–*0 ppm	**0**	30	0	0			
	**1**	20	20	0			
	**2**	50	50	80	3/10	6/10	8/10
	**3**	0	30	20			
	**4**	0	0	0			
	**5**	0	0	0			
							
Infected*–*5 ppm, 30 min	**0**	50	0	0			
	**1**	40	30	20			
	**2**	10	30	60	5/10	4/10	4/10
	**3**	0	30	20			
	**4**	0	10	0			
	**5**	0	0	0			
							
Infected*–*10 ppm, 15 min	**0**	30	0	10			
	**1**	20	0	0			
	**2**	50	67	60	4/10	5/10	6/10
	**3**	0	33	30			
	**4**	0	0	0			
	**5**	0	0	0			

Percentage distribution of microscopic scores of AGD pathologies in the gills of PAA-treated fish. The presence of amoeba in the histological section was, likewise, evaluated and given as *number of samples where the parasite is present/total number of samples analyzed.

**Figure 7:**
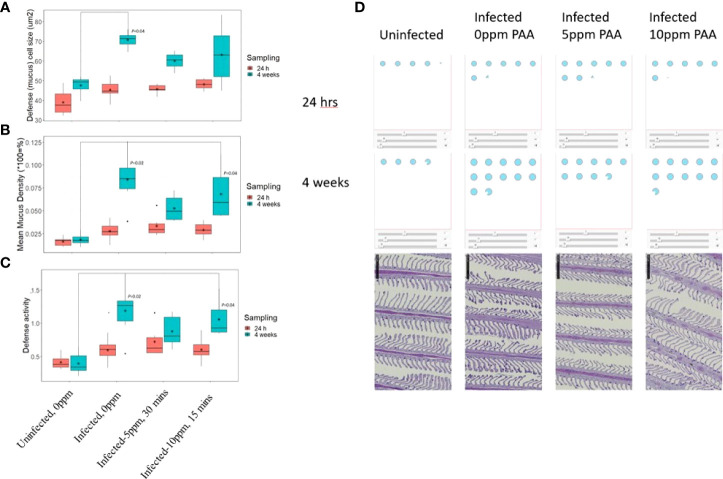
Features of mucous cells in the gill lamellae of AGD-affected Atlantic salmon treated with PAA. **(A)** The mean mucous cell size at the cell equator (µ2) and **(B)**, the mean mucous cell volumetric density (%) in the gill lamellar epithelium and **(C)** Defense activity calculated as (1/Area : Density)x1000. Measurements were taken at 24 h and 4 weeks after treatment. Significant differences with P-values are indicated above the relevant boxplots. N= 6 per fish/treatment group. **(D)** Illustration of the mucous cell size, density, and defense activity in a standardized 100 x 100 µ2 epithelium (Dicer App v2) in the gill lamellae of Atlantic salmon in a Control group (uninfected) or exposed to AGD and treated with either 0-ppm, 5-ppm, or 10-ppm PAA. The blue dots represent the mean mucous cell values per group and per time. The bottom row shows sections of a representative gill from each group after 4 weeks. Note that the patchiness of the mucous cell distribution of the entire gill area is standardized through the application of the Dicer.

**Figure 8:**
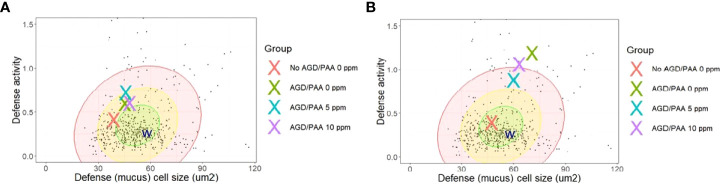
Comparison of group mean mucosal parameters of the gill lamellae (respiratory surface) of AGD-affected, PAA-treated Atlantic salmon relative to the database of Quantidoc of farmed Atlantic salmon of similar weight (n=524). **(A)** 24-h post-exposure. **(B)** 4 weeks post-exposure. Defense activity is calculated from the equation (1/(A:D)) x 1000 where A is mucous cell size and D is volumetric density in the epithelium. The W represents the mean values of gills of seven wild adult salmon captured with permission. The individual dots are individual values from the database of Quantidoc. N= 6/group, N= 48.

Mucosal Mapping revealed that at 24 h after treatment, mucous cell size and density did not significantly change among the treatment groups, although the group means were separating (**Figures 7A,B**). However, after 4 weeks, infection with AGD led to a significant increase in mucous cell area, volumetric density, and defence activity relative to the uninfected group (**Figure 7**). The infected but untreated group (AGD, no PAA) had the mean largest cells at the mean highest density and was clearly in the vulnerable zone of the database of Quantidoc (**Figure 8**). Increasing the treatment dose to 10 ppm PAA increased the mucous cell density and defence activity without significantly affecting the cell size. The variation in the individual response was more apparent in “infected–10 ppm, 15 min” group relative to the “uninfected–0 ppm”.

Comparing the changes in the mucous cell parameters to the database of Quantidoc, which included 524 specimens within the same weight range, the uninfected–0 ppm group showed mean gill mucous cell values, which were near the mean of generally healthy salmon of the same size range (**Figure 8A**). After 4 weeks, this treatment difference increased and the infected groups, regardless of the treatments, were all in the “red zone” or beyond (**Figure 8B**).

### 3.7 Responses to a secondary stressor

**Figure 9:**
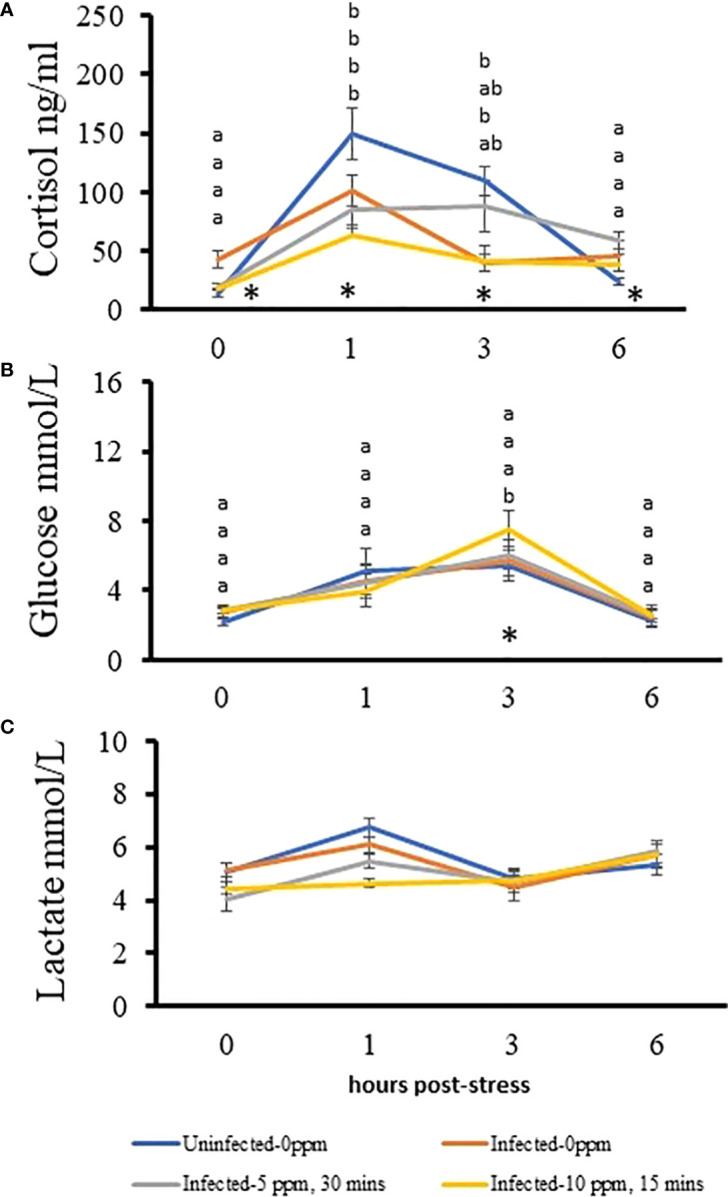
Changes in the plasma stress indicators after subjecting AGD-affected, PAA-treated Atlantic salmon to crowding. A stress test was performed after the last sampling. The levels of **(A)** cortisol, **(B)** glucose, and **(C)** lactate were measured in 10 fish per treatment group at each timepoint. The significant difference over time in a treatment group is indicated with different letters. Groups are arranged from top to bottom: uninfected–0 ppm; infected–0 ppm; infected–5 ppm, 30 min; and infected–10 ppm, 15 min. An asterisk (*) indicates that a significant inter-treatment difference exists among the groups at that particular timepoint, which has been detailed in the text.

Plasma cortisol level significantly increased in all groups 1 h after stress and returned to basal level after 6 h (**Figure 9A**). The “uninfected–0 ppm”, “infected–0 ppm” and “infected–10 ppm, 15 min” showed almost an identical patterns of cortisol response after stress, though the magnitude was higher in “uninfected–0 ppm”. This was reflected in timewise inter-treatment differences especially the level of cortisol in “uninfected–0 ppm” group which was significantly higher than the infected-treated groups at 1 and 3 h after stress. It was also observed that at time 0 (pre-stress), the cortisol level in “infected–0 ppm” group was significantly higher than the rest of the groups. On the other hand, cortisol level, 6 h post-stress in “uninfected–0 ppm” group, was significantly lower than the other groups. Only the glucose level in “infected–10 ppm, 30 min” group showed significant variations after stress, where it peaked at 3 h after stress induction and returned to pre-stress level at 6 h after stress (**Figure 9B**). Moreover, its level at this timepoint was significantly higher than the “uninfected–0 ppm” group. The plasma lactate level does not change after stress induction (**Figure 9C**).

## 4 Discussion

Our understanding of the epidemiology and pathophysiology of amoebic gill disease (AGD) has progressed dramatically in the last years—from documentation of gross and microscopic pathologies, which have already been routinely used for disease assessment to the molecular aspects of host-parasite interactions that identify biomarkers for potentially novel diagnostic assays (16, 38). However, there is a paucity of information on the physiological and immunological consequences of potential chemotherapeutics in AGD-affected fish. Poor gill health is increasingly implicated in a variety of disease complexes that may be exacerbated by rough husbandry manipulations such as the delousing; in Norway alone, over 2,983 delousing were performed in 2020 (15). Latent tissue-wide response of up to 2–3 weeks post-stress in gills (12, 35) has made preventative measures for AGD challenging to identify. Here, we showed that exposing AGD-affected fish to PAA, an oxidizing agent with strong biocidal activity, influenced the physiological and immunological responses to the infection and offered opportunities for improvement to develop new candidate chemotherapeutics.

### 4.1 PAA treatment may pose health and welfare risks to AGD-affected salmon

Though often regarded as one of the most eco-friendly disinfectants in aquaculture, identifying the safe dose of PAA is often presented with a challenge because the toxicity window for many fish species, including Atlantic salmon, is narrow (29, 39). The doses used in the present study were identified in a series of studies where naïve Atlantic salmon had been exposed to PAA at different doses, duration, and frequencies (31, 32). Mortality was documented in “infected–10 ppm, 15 min” shortly after exposure, and the survival level 24 h after treatment was lower than the other exposed groups. Lesions of lamellar vessels and lamellar adhesions were identified in PAA-treated fish, which is likely an additional factor that aggravated the impact on the respiratory function of AGD-affected fish. This welfare-related issue must be considered in using PAA as a chemotherapeutic. It was also apparent that the infected groups, regardless of the PAA treatment administered, appeared to be more susceptible to PAA toxicity. Such a response was not observed in the uninfected groups, as well as in previous PAA trials where these doses had been investigated and identified (31, 32). This suggests that AGD might have lowered the tolerance threshold of Atlantic salmon to PAA, thereby increasing the toxicity risk. This hypothesis is further supported by the cellular responses to the treatments: while the uninfected group had the smallest and fewest cells typical of healthy gills, infestation with AGD produced many and large mucous cells commensurate with quite vulnerable gills (**Figure 8**). Unfortunately, increasing the dose to 10 ppm PAA induced more but not larger mucous cells in the gills. These results support a balance between mucosal protection from infection or exposure to toxicants and the effective dose of PAA or other therapeutic actions, as has been found in previous studies (35, 40). This could be related to the dysregulation in immunity of AGD-affected fish (38), limiting their adaptive capacity to the oxidant. The magnitude of behavioral changes in the infected groups was, likewise, greater, thus, lending support to the hypothesis that the infection might have increased the sensitivity of salmon to PAA. These results provide another dimension on PAA toxicity in Atlantic salmon and, thus, must be considered in the informed basis of its application.

### 4.2 Plasma indicators reveal the effects of AGD on internal organ health

Respiratory disturbances associated with the proliferation and fusion of the lamellar epithelium in AGD-affected fish decrease the functional gill surface area and increase the diffusion distance in the water-blood barrier for oxygen transfer, which will result in secondary hypoxic changes in the liver (11, 41). Here, we documented that liver health was affected by AGD, as indicated by the increase in plasma level of alanine transaminase (ALAT) and alkaline phosphatase (ALP). Higher levels of these analytes are often implicated in liver damage. These analytes were elevated 24 h and 4 weeks after treatment in the “infected–0 ppm” group indicating secondary effects of gill infection on liver function. At 4 weeks post-treatment, ALAT showed that PAA treatment might have protected the liver from AGD-associated damage, but ALP suggested otherwise. It would be interesting to evaluate in the future the specificity of these analytes to differentiate the infection severity of AGD. In addition, clinical biochemistry in fish is often presented with the challenge of the lack of standards on differentiating health status. Tissue leaks LDH when damaged (42). The elevated level of plasma LDH is most likely connected to structural alterations in the gills that are associated with the pathology of AGD, which might have released LDH into the bloodstream. Interestingly, the level did not increase following PAA treatment, indicating that the oxidant did not intensify the organ damage associated with AGD. This was supported by histology. It was previously identified that at the concentrations tested, PAA caused only minor reversible gill alterations (31, 32). Systemic indicators for AGD are not commonly explored; therefore, the changes in LDH present a potential biomarker for AGD that should be validated in future infection studies.

### 4.3 Infection induces systemic oxidative stress but is not exacerbated by PAA treatment

Oxidative stress, the imbalance of reactive oxygen species and the inability of the organism to scavenge and neutralize them, has been linked to the pathophysiology of many diseases (43). It is known that PAA is a mild environmental stressor, and its ability to trigger transient systemic and mucosal oxidative stress has been thoroughly documented in Atlantic salmon smolts (30, 33). The results demonstrated that AGD alone or in combination with PAA treatment induced systemic oxidative stress by increasing the level of ROS (arbitrarily measured as H_2_O_2_) in plasma. The alteration of oxidative stress status has been implicated as a key mechanism at the later stage of AGD (44). The significantly elevated ROS level observed 4 weeks after treatment when gill scores were between 2 and 3, corroborated the earlier evidence of the involvement of oxidative stress in the pathophysiology of AGD. Increased production of ROS during infection facilitates pathogen clearance and contributes to signaling cascades related to inflammation, cell proliferation, and immune responses (45), and very likely, these mechanisms are at play as well in AGD. Treatment with PAA did not increase the ROS level indicating that the oxidative stress-inducing ability of AGD was not influenced by another strong oxidative chemical stressor.

At the mucosal level, several regulators of oxidative stress genes have been identified in the gill transcriptome and, in most cases, the rate of transcriptional change was higher at the early timepoints. For example, the expression of *thioredoxin reductase 3*, *glutathione peroxidase 1*, and *superoxide dismutase* genes involved in ROS scavenging and detoxification (30), were highly affected 24 h after treatment. It was also apparent that infected groups treated with PAA had a higher magnitude of change than the infected–0 ppm. This demonstrates that the known consequence of PAA as an inducer of transient oxidative stress (32, 46) was magnified by parasitic infestation. The changes in the expression of key oxidative stress-related genes in the gills and the alterations in systemic ROS level underscored a salient pattern that mucosal oxidative stress is triggered as an early phase response followed by systemic oxidant-antioxidant dysregulation as a subsequent consequence during the later stage of infestation.

Fish have an array of antioxidant molecules that ensure redox homeostasis and play a crucial defense role during oxidative stress (47). At 4 weeks post-treatment, plasmatic TAC significantly increased, such as the elevated level of ROS level in AGD-infected, PAA-treated fish. This provides evidence that AGD and PAA treatment did not impede the inherent ability of salmon to counteract elevated ROS. We have earlier shown that salmon has an efficient antioxidant system that addresses oxidative stress triggered by oxidative chemotherapeutics (32, 46). Interestingly, in the early timepoints, there was a variability in the TAC level, particularly with the “infected–10 ppm, 15min” group. This implies that the immediate and mid-recovery TAC responses could be influenced differentially by the high PAA dose, suggesting a slight interference with the systemic antioxidant system following treatment.

### 4.4 PAA modifies the gill transcriptomic response to AGD, but predominantly shortly after treatment

Several transcriptomics studies on AGD, either by qPCR assay of a large panel of genes, microarray or RNA sequencing, have elucidated the molecular mechanisms associated with the onset of the disease and its progression (5, 16, 38). However, many of the available transcriptomic datasets vary depending on the dose and virulence of the *N. perurans*, fish size, tank environment, duration of infection, and use of lesion or non-lesion specific gill tissue, which often pose a challenge when performing comparisons. Nonetheless, there are several mechanisms that have been implicated that likely govern host-pathogen interaction from the onset until the later stage of the disease. To our knowledge, this is the first study that explored transcriptome-wide responses of AGD-affected fish after treatment. It is evident in the gill transcriptome data that PAA treatment of AGD-affected fish had stronger effects immediately after treatment and the infected groups (treated and untreated) had almost the same transcriptomic response thereafter. This suggests that the modulation of response to AGD by the oxidant was only transitory and did not persist during the recovery/disease progression phase. This can partly explain why gross and microscopic pathologies were almost similar among groups at 4 weeks post-treatment, though some changes were distinguished by the Mucosal Mapping.

There were four main clusters—clusters 2, 3, 4, and 5 that conspicuously showed that oxidant treatment altered the transcriptomic responses of AGD-affected fish at 24 h after treatment. In Cluster 2, proteases involved in immunity and metabolism were substantially affected and characterized by upregulation in the PAA-treated groups where the magnitude of change was dose-dependent. The *mmp 13* is expressed in wound keratinocytes and may be stimulated by the small ROS, hydrogen peroxide (48). Given that H_2_O_2_ is one of the constituent components of the PAA trade product, the upregulation of several *mmp 13* genes may likely be a response to the oxygen radical. Moreover, this lends support to the discussion earlier that mucosal oxidative stress was triggered after treatment. In addition, the upregulation of *heat shock protein 90, alpha* (*hsp90a*), especially at the highest dose, suggests stress responses in the gills. This group of genes indicate that stress might have been triggered at the gill mucosa by PAA treatment but not considerably by AGD.

It was reported earlier that AGD-affected gills displayed an increased mRNA expression of cellular markers of immune cells, including professional antigen-presenting cells (*mhc-ii, cd4*), B cells (*igm, igt, mhc-ii*), and T cells (*tcr, cd4, cd8*) (19). In another study, a coordinated down-regulation of MHC I pathway-related genes during the later stages of infection was demonstrated, including the down-regulation of *interferon-regulatory factor* (*irf*)-1, *independent of interferon-α*, *interferon-γ*, and *irf-2* expression (18). In Cluster 3, we found an overrepresentation of genes responsible for T- and B-cell–mediated immune response, where the infected groups showed downregulation at 24 h after treatment and, interestingly, the impact was magnified by oxidant treatment. The group receiving 10 ppm PAA for 15 min showed the highest downregulation among the groups. In earlier PAA exposure studies, we did not find a strong impact of PAA on these molecules (31, 49). This indicates that PAA may impair B- and T-cell–mediated response only when there is a pre-existing infection. Furthermore, such a weakening response triggered by PAA likely contributed to an environment that allows continuous invasion and proliferation of the parasite. It was also interesting to document that B- and T-cell–mediated response appeared to be marginal at 4 weeks post-infection, where a number of fish already exhibited gill scores >2. This is partially contradictory to the earlier observation on the role of these molecules during the later stage of infection (19). These results present another point for consideration in the ongoing discussion on how to devise the best approach to unravel the mechanisms behind complex disease states such as AGD (38).

Oxidative stress-inducing compounds, such as H_2_O_2_ and PAA, require robust metabolic programming from the host to ensure efficient deployment of response for homeostasis and adaptation (49, 50). Cluster 4 showed genes mainly involved in metabolism in mitochondria, RNA and protein, where their expression was upregulated in infected-PAA–treated groups at 24 h after treatment. Moreover, the change was greater in the group that received 10 ppm PAA for 15 min. We can speculate that this substantial change may be involved in repairing cellular damages induced by compound stressor oxidant and AGD. One of the genes, which the rate of change was quite large in “infected-10, 15 min” group, was *protein arginine N-methyltransferase 3* (*prmt*), a gene responsible for methylating arginine residues in histone and non-histone proteins and has a major role in transcription and chromatin regulation, cell signaling, DNA damage response, RNA and protein metabolism, and stress (51). This points to its potential involvement in the response to the high-oxidant dose. GTPases are associated with diverse cellular processes, including signal transmission, cell polarity, cell cycle progression, gene expression, material transport, and construction of the cytoplasmic skeleton (52). We have identified a significant subset of GTP-related genes that were upregulated in PAA-treated groups 24 h after treatment, including *GTPase activating protein*, *guanine nucleotide-binding protein-like 3*, and *GTPase IMAP family member 7*. It has been reported previously that GTPases in the mucosal organs of salmon were affected by PAA, where its regulation following exposure has been implicated in facilitating radical scavenging (31, 49). The results concur that these molecules may be involved in the response to oxidant PAA, and this crucial function was not interfered with by the disease state.

Impairment of immune response in AGD has been documented in several studies, which likely contributes to the successful attachment, invasion, and proliferation of the parasite on the gill mucosa (11). This was exhibited in the gill transcriptome of AGD-affected fish as shown by Cluster 5, where most immune-related genes involved in chemokine, complement, and Ig-mediated responses clustered, and treatment-related differences were more notable at 24 h after treatment. Cytokines in the Th1, Th2, and Th17 cell differentiation pathways are often considered to be involved in AGD immune response, where downregulation is often a hallmark response for Th1, Th17, and Tregs pathways, whereas pro-inflammatory cytokines (Il-1β) and Th2 cytokines exhibit upregulation (3, 20). We have identified *interleukin 1b* (including the receptor) and several interferons to be downregulated mostly all throughout the trial in infected–0 ppm, as well as in two PAA-treated groups. Downregulation of these important cytokines could have facilitated the progression of the disease.

Increased mucus production is commonly associated with AGD (11), which was also identified in the current study through mucosal mapping. The AGD-affected fish began to change the mean mucous cell sizes within 24 h after treatment. At the same time, the uninfected control group showed mean gill mucous cell values were near the mean of generally healthy salmon of the same size range. After 4 weeks, this treatment difference increased and the infected groups exhibited activated protection through both hyperplasia and hypertrophy of the mucous cells (**Figure 8B**). This agrees with commonalities for complex gill histopathology, where epithelial and mucous cell hyperplasia were two of the three most frequent findings (53). Thus, the infection with AGD likely set the gill barrier cells on a pathway toward active chronic mucosal protection, which was alleviated by effective treatment with 5-ppm PAA but only somewhat ameliorated by the 10-ppm dose of PAA. In particular, *mucin 5* (*muc5*) is most likely the main component of the characteristic mucus patches comprising AGD lesions (16). We have identified *muc5* in the gill transcriptome to be heavily affected by AGD, where its expression increased as the disease developed, thus lending support to an earlier proposed involvement in AGD. Another mucus-associated gene that demonstrated a higher magnitude of change in response to infection and treatment was *GMP Giant mucus protein* (*gmp*), a high-molecular weight multi-domain protein specific for fish and responsive to ectoparasite salmon louse (54). This gene was earlier shown to be affected by PAA and was implicated to be involved in mucosal protection from the chemical oxidative stressor (46).

### 4.5 Oxidant treatment can reduce the parasite load, but pathologies are still persistent

The spectrum of antimicrobial activity of PAA is well documented, including against relevant bacterial and parasitic pathogens in aquaculture (55–57). Under *in vitro* conditions, PAA exhibit amoebicidal activity against *N. perurans* within the concentrations tested in this study (unpublished). PAA is in the family of oxidizing agents where H_2_O_2_, the most well-known oxidant treatment for AGD, belongs. We evaluated for the first time the potential of PAA as a chemotherapeutics against AGD-affected salmon. The gills of AGD-affected fish exhibited classic gross pathologies such as white mucoid patches (8) and were supported microscopically by evidence of multifocal hyperplasia and lamellar fusion (10). Moreover, the parasites were, likewise, observed and detected in the tissue sections, indicating that the infected fish used represented diseased specimens. In addition, the number, size, and volumetric density of mucus cells in the gills increased in AGD-affected fish, which supports earlier evidence of increased mucus production during infestation (16).

From gross gill scoring, PAA treatments did not reduce the pathologies associated with the disease. The frequency of gross gill score 2 and higher increased as the recovery period progressed, and the distribution of scores showed slight differences among groups. Interestingly at termination, microscopic scoring revealed that the percentage of the population having an AGD pathology score >2 was the highest in “infected–0 ppm”, whereas the lowest in “infected–5 ppm, 30 min”. Though this subjective difference between groups were not large, it agreed with the sensitive Mucosal Mapping results and was supported by qPCR quantification where the lowest parasite load was detected in “infected–5 ppm, 30 min”. Therefore, the treatment of AGD-affected fish with PAA resulted in equivocal disease resolution; nonetheless, 5-ppm PAA for 30 min could reduce the parasite load and may, likely, require time for the pathologies to recover. It is known that fish will still have high gill gross score when they are recovering, which likely explains the contrast between the parameters for assessing disease resolution in the timeframe of this study. In earlier treatment experiments, it was demonstrated that the efficacy of H_2_O_2_ against AGD was not highly dose-dependent (9); somehow, PAA as a treatment followed such a trend. Moreover, H_2_O_2_ treatment does not cure the fish, but delays the development of the disease and growth of the amoeba (9, 22, 24), despite tissue-wide persistent disruption of barrier mucosa (12). A longer period of recovery after treatment is required for us to verify whether such consequences are also true for PAA.

### 4.6 AGD and PAA treatments alter the kinetics of stress response to a secondary stressor

We have shown earlier that frequent exposure to PAA did not affect the ability of salmon smolts to respond to a secondary stressor, but slight modifications in the kinetics of response were documented (32, 33). For example, fish exposed to PAA had a higher cortisol response than unexposed fish. In the present study, we found that AGD and PAA treatment did not impede the ability of salmon to respond to a secondary stressor; however, the magnitude and, to some extent, the kinetics, had been altered. It appears that AGD dampened the ability to mobilize cortisol after stress because infected groups showed lower cortisol responses. As an anti-inflammatory molecule, cortisol participates in the activation of immune response (58), and is very likely active in resolving the AGD-related dysregulation of the host immunity, as shown in the gill transcriptome of infected fish. The lower magnitude of cortisol response in the infected group suggests potential exhaustion, in which cortisol had been extensively utilized for immune regulation as the disease progressed, thus, a lesser response was demonstrated when secondary stress is encountered. Moreover, fish exposed to 10 ppm for 15 min showed the lowest cortisol levels among the groups post-stress, indicating a potential compound interference caused by infection and oxidant on cortisol mobilization. Cortisol has been found to be elevated in AGD-affected fish (59), as well as in PAA exposed salmon (32). We documented that “infected–0 ppm” had significantly higher cortisol levels before stress induction, which suggests chronic stress in this group. Chronic stress from elevated cortisol may interfere with immune responses (60), and this partly explains why in the cluster of immune genes in the gill transcriptome, the responses in the infected untreated group were marginal.

Glucose and lactate are involved in the secondary response to stress and play a key role in energy metabolism and allocation (61). The treatment groups did not show significant changes in both parameters after stress induction except in “infected–10 ppm, 15 min” where the glucose level increased at 3 h after stress. Though it is difficult to provide a solid implication of the physiological importance of such a treatment-specific response, providing the compound stressor may slightly magnify the ability of fish to mobilize glucose reserves following stress.

## 5 Conclusions

The present study revealed new insights into the physiological consequences of AGD and how they were influenced by oxidative chemotherapeutics. PAA treatment of AGD-affected fish increased the toxicity risk of PAA, hence, providing a crucial aspect that must be thoroughly considered in using this oxidant as a chemotherapeutics. AGD interfered with liver functions and induced systemic oxidative stress, nonetheless, treatment with an oxidant did not aggravate the AGD-induced alterations. These results further expanded the role of oxidative stress in the pathophysiology of AGD. The gill transcriptome elucidated the molecular changes following infection and treatments, revealing how mucosal cellular and humoral immune responses were orchestrated in response to infestation, and, in some cases, modulated by PAA treatment at a higher dose but shorter exposure duration. It was apparent that PAA treatment could interfere with gill transcriptomic responses in AGD-affected fish, but only shortly after treatment. PAA treatments did not fully resolve the disease-associated pathologies as gill scores further developed during recovery, although mucosal mapping measures indicate that AGD, either treated or untreated, displayed significantly larger and denser cells than in uninfected fish gills and may be useful in the early detection of infections and to identify effective therapeutic interventions. Despite the equivocal treatment impacts on gross and microscopic pathologies, we documented lower parasite load in the group, 5 ppm, 30 min group. The delayed or latent responses of tissues, and the lack of correspondence between gill scores and other pathologies, underscore the need to have more objective analytical methods to assess disease state and resolution. AGD and PAA treatments did not impede the ability of fish to respond to secondary stress; however, infection and treatment altered the magnitude and kinetics of response, indicating a potential interference of the stress axis. Lastly, the study offered new insights into host–pathogen–treatment interactions in AGD research. PAA as a treatment for AGD requires further investigation, mainly focusing on identifying the optimum treatment protocol. Nonetheless, the results presented here would be valuable in the evidence-driven use of PAA as an aquaculture disinfectant in general.

## Data availability statement

The data presented in the study are deposited in the GEO repository under accession number GSE211350.

## Ethics statement

This study was reviewed and approved by Norwegian Food Safety Authority (FOTS ID 20/37233).

## Author contributions

*Funding*: CL and L-FP. *Conceptualization*: CL, L-FP, MB, SH, and KP. *Planning*: CL, MB, and FF. *Execution of trial*: CL, MB, DS, and FF. *Sampling*: CL, MB, FF, and DS. *Laboratory analysis*: CL, LP, GM, DS, and MG. *Data analysis and curation*: CL, AK, GT, GM, KP, DS, and MG. *Interpretation*: CL, FF, MG, GT, and KP. *Visualization and presentation*: CL, GT, and GM. 1^st^ draft *Writing*: CL. *Editing and Final Draft*: All. All authors contributed to the article and approved the submitted version.

## Funding

This study received funding from the Norwegian Seafood Research Fund (ref. 901472, Peragill).

## Acknowledgments

We thank the personnel at the Fish Health laboratory of HiT for their technical assistance during the fish trial. We also acknowledge the assistance of Marianne H. S. Hansen in the microarray analysis (Nofima). The chemical used in the trial was provided by Lilleborg (Dr. Lisbeth Rørmark). F F wishes to acknowledge the ERASMUS+ Student Mobility Programme for financing his research stay at Nofima. We would like to thank Dr. Linda Andersen of the Industrial and Aquatic Laboratory (ILAB) for her insightful comments to this paper.

## Conflict of interest

KP and GM work for Quantidoc AS.

The remaining authors declare that the research was conducted in the absence of any commercial or financial relationships that could be construed as a potential conflict of interest.

## Publisher’s note

All claims expressed in this article are solely those of the authors and do not necessarily represent those of their affiliated organizations, or those of the publisher, the editors and the reviewers. Any product that may be evaluated in this article, or claim that may be made by its manufacturer, is not guaranteed or endorsed by the publisher.

## Author disclaimer

Mention of trade names or commercial products in this paper is solely for reporting and does not imply recommendation or endorsement by Nofima, the Technical University of Denmark, the University of Bergen, the University of Lisbon, and the Norwegian Veterinary Institute.

## Supplementary material

Supplementary Table 1 List of all differentially expressed genes.
